# 

**Published:** 1899-10-28

**Authors:** 


### NOTICE TO CORRESPONDENTS.

All MS., Letters, Books for Review, and other matters intended for the Editor should be addressed The Editor, "The Hospital," The Hospital Building, 28 & 29, Southampton Street, Strand, W.C.

The Editor cannot undertake to return rejected MS., even when accompanied by stamped directed envelope.

**Notice to Advertisers and Subscribers.**

All Advertisements, Orders for Copies of The Hospital, and Business Communications should be addressed to **THE MANAGER (Not to the Editor)**, The Hospital, The Hospital Building, 28 & 29, Southampton Street, Strand, London, W.C.

The Cost of Subscription, post free, is as follows:—Per week, 2½d.; per quarter, 2s. 8d.; per half-year, 5s. 3d.; per annum, 10s. 6d. Foreign:—Per annum, 15s. 2d., payable, in all cases, in advance.

**NOTICE.—Vol. XXVI. of "The Hospital" (April, 1899, to September, 1899), in handsome cloth binding, will shortly be on sale at the Office of this Journal, price 7s. 6d. Cases for binding the Numbers contained in this Volume 1s. 6d. Index to Vol. XXVI., 2½d., post free.**

**The Monthly Part for November will be ready on November 1st. Price 6d., by post 8d.**

### Scraps and Gleanings.

One thousand seven hundred and forty pounds is still required to defray the expenses of the building operations which are being carried on at the North Riding Infirmary.

The Combe Lying-in Hospital (Dublin) appears to have done considerable work during the past year. With the Weir bequest of £6,000 the chronic department is to be considerably altered, and steps are to be taken to establish some memorial to perpetuate the name of the donor of this handsome bequest. A nursing institution in connexion with the hospital is also to be established, thanks to the gift from the Countess of Meath of £250.

The Jubilee Convalescent Home Fund for Bristol is now within £7,000 of the £100,000 required for the endowment fund. This £7,000 must be provided by the beginning of November, otherwise several promises to give sums amounting to £8,000 will lapse, as these sums were only promised conditionally on the total sum being provided.

The last quarterly report of the Norfolk and Norwich Hospital stated that unless the income were promptly increased one of the wards would have to be closed. Already during this year the board has had to sell out £3,000 of their invested property, the income having declined from £3,956 in 1885 to £2,945 in 1898, and there is now an annual deficiency of £2,000 between the ordinary income and the ordinary expenditure. To set the finances of the hospital on a proper footing a sum of £9,500 is required.

### The Student of Medicine.

### APPOINTMENTS.

B. Addenbrooke, M.D., B.S., M.B., B.Hy., M.R C.S.Eng., L.R.C.P.Lond., appointed Honorary Surgeon to the Kidderminster Infirmary and Children's Hospital. E. H. Addenbrooke, M.R.C.S.Eng., L.S.A., appointed Honorary Consulting Surgeon to the Kidderminster Infirmary and Children's Hospital. R. T. Bailey, M.R.C.S.Eng., L.R.C P.Lond., appointed Resident Medical Officer to the Ashton Street Department (Lock) Royal Infirmary, Liverpool; and House Surgeon to the Ophthalmic, Laryngological, and Aural Departments, Royal Infirmary, Liverpool. J. M. Beattie, M.A., M.B., C.M.Edin., appointed University Tutor in Clinical Medicine, Royal Infirmary, Edinburgh. W. Davidson, L.R.C.P., L.R C.S.Edin., appointed Medical Officer for the Sixth District of the Beverley Union. J. Evans, M.R.C.S Eng., appointed Medical Officer of the Children's Homes of the Cardiff Union. W. Guy, F.R C.S., L.R.C.P., L.D.S Edin., appointed Dean of the Edinburgh Dental Hospital and School. R. Hanton, M.B., C.M.Edin., appointed Medical Officer for the West Hartlepool and Stranton District of the Hartlepool Union. E. Hutton, M.R.C.S Eng, L.R.C.P.-Lond., appointed Visiting Surgeon to the Stockport Infirmary. J. Luckhoff, M.B., Ch.B., appointed a Clinical Assistant to the Chelsea Hospital for Women. H. C. Moore, M.R.C.S.Eng., L.S.A., appointed Medical Officer of Health to the Hereford Town Council. J. C. Muir, M.B., B.C.Camb., appointed Second Resident Assistant Medical Officer at the Crumpsall Workhouse, Manchester. C. P. Oliver, M D Lond., D.P.H., appointed Medical Officer of Health to the Maidstone Urban Rural District Council. A. F. Perigal, M.B., Ch.B., appointed a Clinical Assistant to the Chelsea Hospital for Women. G. Pernet, M.R.C S.Eng., L.R.C.P.Lond., appointed Pathologist to the Hospital for Diseases of the Skin, Stamford Street, Blackfriars. A. Quennell, M.R.C.S., L.R.C.P.Lond., appointed Medical Officer for the Mountnessing District of the Billericay Union. H. E. Rayner, F.R.C S.Eng., appointed Medical Officer of the Frimley District of the Farnham Union. J. A. Reed, M.B., Ch.Vict., appointed House Surgeon to the Stockport Infirmary. A. K. Reede, L.R.C.P.Edin., L.M., L R C.S.Edin., appointed Medical Officer of the Yarcombe District of the Honiton Union. W. Scatterty, M.D.Aberd., appointed Medical Officer of Health to the Keighley School Board. A. T. Sisson, M.B., B.Ch., appointed Junior Resident Assistant Medical Officer at the Crumpsall Workhouse of the Township of Manchester. E. Trotter, M.B., Ch B., appointed House Surgeon to the Hospital for Women and Children, Leith. E. E. Willis, L.R.C.P.Edin., L.R.C.S.Edin., L.F.P.S.Glasg., appointed Medical Officer of the Seventh District of the Downham Union.

Leith Hospital.—The following appointments to the Resident Medical Staff have been made:—House Physician: Edith Hudson, M.B., C.M.; House Surgeon: A. D. Yule, M B., C.M., B.Sc.; Assistant House Physician: Harriet A. Bird, M.B., Ch.B.; Assistant House Surgeon: J. C. Carr M B., Ch.B.; Surgeon in the Outdoor Department: Robina McGregor, M.B., Ch.B.

### Medical Appointments.

LONDON HOMŒOPATHIC HOSPITAL,

GREAT ORMOND STREET, W.C.

A VACANCY occurs for a RESIDENT MEDICAL OFFICER, the appointment being for twelve months, six months as House Surgeon and six months as House Physician, with salary at the rate of £80 per annum, and Board and Apartments.

Candidates must be legally qualified, and be, or become, members of the British Homœopathic Society.

Applications, with twenty-five copies of testimonials, may be sent in to the Secretary-Superintendent as early as possible.

(878) G. A. CROSS, Secretary-Superintendent.

LONDON HOMŒOPATHIC HOSPITAL,

GREAT ORMOND STREET, BLOOMSBURY.

The Board of Management are prepared to receive APPLICATIONS for APPOINTMENT to the POST of ANÆSTHETIST.

Honorarium £50 per annum.

Applications to be sent in not later than November 20th to the Secretary-Superintendent, of whom further particulars may be obtained.

(877) G. A. CROSS, Secretary-Superintendent.

WANTED, for the MEDICAL and SURGICAL
CONSULTATIVE INSTITUTION, BIRMINGHAM.

There are VACANCIES for a PHYSICIAN and a SURGEON.

Requisites in either case are a high diploma from one of the Colleges of England, Scotland, or Ireland, and hospital and other experience. Age about 30. Minimum earnings of £500 per annum will be guaranteed for each post, under an agreement for three years, during which private practise will be debarred.

Applications should be addressed to the Secretary, Medical and Surgical Consultative Institution, 7a, Newhall Street, Birmingham, and should be accompanied by testimonials, list of honours, and short account of past experience. (863)

Royal 8vo., cloth gilt, illustrated by a series of Original Photo-micrographs and many Illustrations in the Text, 7s. 6d. net,

THE MENOPAUSE AND ITS DISORDERS.

With Chapters on Menstruation. By A. D. LEITH NAPIER, M.D., M.R.C.P., late Editor of the *British Gynæcological Journal.*

"Of great practical use to the general practitioner as well as to those more specially engaged in gynæcological work."—*The Hospital.*

London: The Scientific Press, Ltd., 28 & 29, Southampton Street, Strand, London, W.C.

A NEW TEXT-BOOK FOR STUDENTS.

Crown 8vo., nearly 150 pp. Illustrated. Cloth. Price 2s. 6d.

**MEDICAL GYMNASTICS,** Including the SCHOTT (Nauheim) Movements. Being a Text-Book of Massage and Mechanical Therapeutics Generally, for Medical Students. By AXEL V. GRAFSTROM, B.Sc., M.D. (Late Lieutenant in the Royal Swedish Army; late House Physician, City Hospital, Blackwell's Island, New York.)

London: THE SCIENTIFIC PRESS, LTD., 28 & 29, Southampton Street, Strand, W.C.

**THE UNIFORM SYSTEM OF ACCOUNTS, AUDIT, AND TENDERS, for Hospitals and Institutions, with Certain Checks upon Expenditure; also the Index of Classification.** By Sir HENRY BURDETT, K.C.B. Demy 8vo, profusely illustrated with Specimen Tables, Index of Classification, Forms of Tender, &c., cloth extra, 6s.

**ACCOUNT BOOKS FOR INSTITUTIONS, designed in accordance with the Uniform System of Accounts for Hospitals and Institutions.** By Sir HENRY BURDETT, K.C.B. Being a complete set of Account Books, ruled in accordance with the Uniform System adopted by the Metropolitan Hospital Sunday Fund.
*Full Description of Books and Prices sent post free on application.*

London: The Scientific Press, Ltd., 28 & 29, Southampton Street, Strand, W.C.

### LATEST TEXT-BOOKS FOR NURSES.

Crown 8vo., profusely Illustrated, cloth gilt, 5s.

### A HANDBOOK FOR NURSES.

By J. K. WATSON, M.D., M.B., C.M.

"A concise and comprehensive exposition of as much medical knowledge as a nurse needs. The work cannot but prove useful for whom it has been designed."—*Scotsman.*

---

New Edition, profusely Illustrated, Crown 8vo., cloth gilt, 3s. 6d.

### NURSING: Its Theory and Practice.

Being a complete Text-book of Medical, Surgical, and Monthly Nursing.

By PERCY G. LEWIS, M.D., M.R.C.S., L.S.A.

With entirely new Chapters on Mental Nursing, Mechanical Therapeutics, Massage, Schott, Tallerman, and Weir-Mitchell's Treatments.

"We have read the book with interest, and can warmly recommend it as a nursing manual."—*Birmingham Medical Review.*

---

Crown 8vo., Illustrated, cloth gilt, 7s. 6d. net.

### PRACTICAL POINTS IN NURSING.

For Nurses in Private Practice. By EMILY A. M. STONEY.

"The hints given are excellent, and we hope they may be widely read."—*British Medical Journal.*

---

Crown 8vo., profusely Illustrated, cloth gilt, 6s.

### PHYSIOLOGY: Experimental and Descriptive.

By B. P. COLTON, Professor of Natural Science, Illinois University.

*Complete Catalogue of Works on Nursing and allied subjects sent post free on application.*

LONDON: THE SCIENTIFIC PRESS, LTD., 28 & 29, Southampton Street, Strand, W.C.

### NOW READY,

A REPRINT OF

### A NURSING HANDBOOK

TOGETHER WITH S. MAW, SON, AND THOMPSON'S

### COMPLETE CATALOGUE OF NURSING REQUISITES.

The above work is now republished at a cost of 1s. per copy. Messrs. S. M., S., and T. have been compelled to make this charge on account of the extreme popularity of the work and the great expense incurred in its production.

***Post Free on receipt of 1s.***

Nurses' Bags.

### S. MAW, SON, and THOMPSON,

7 to 12, ALDERSGATE STREET, LONDON, E.C.

